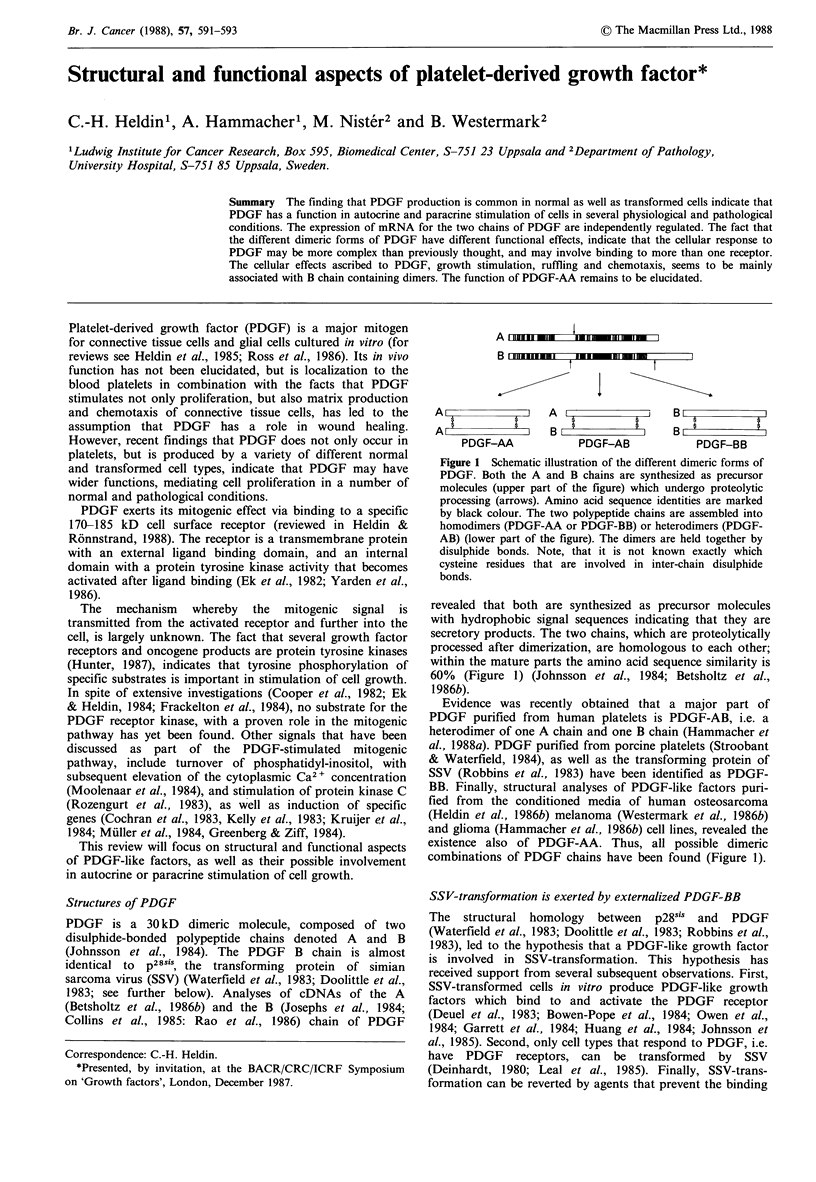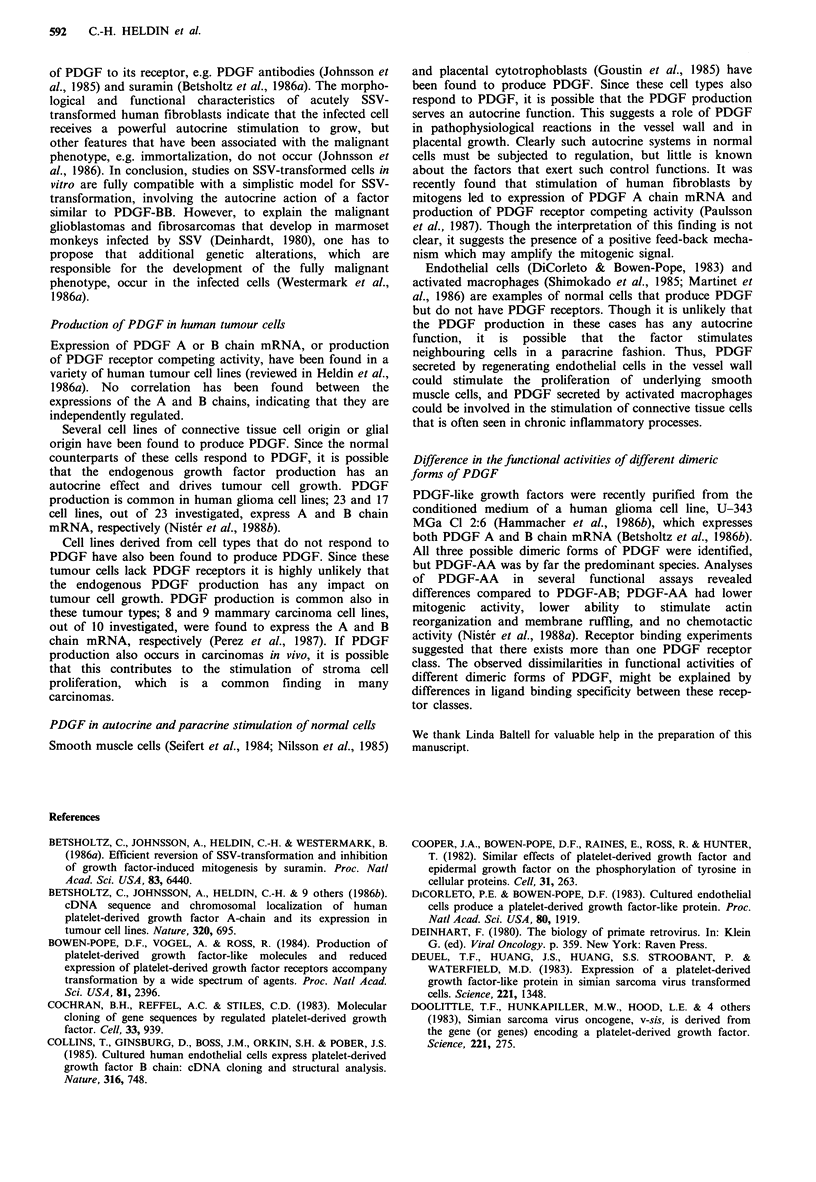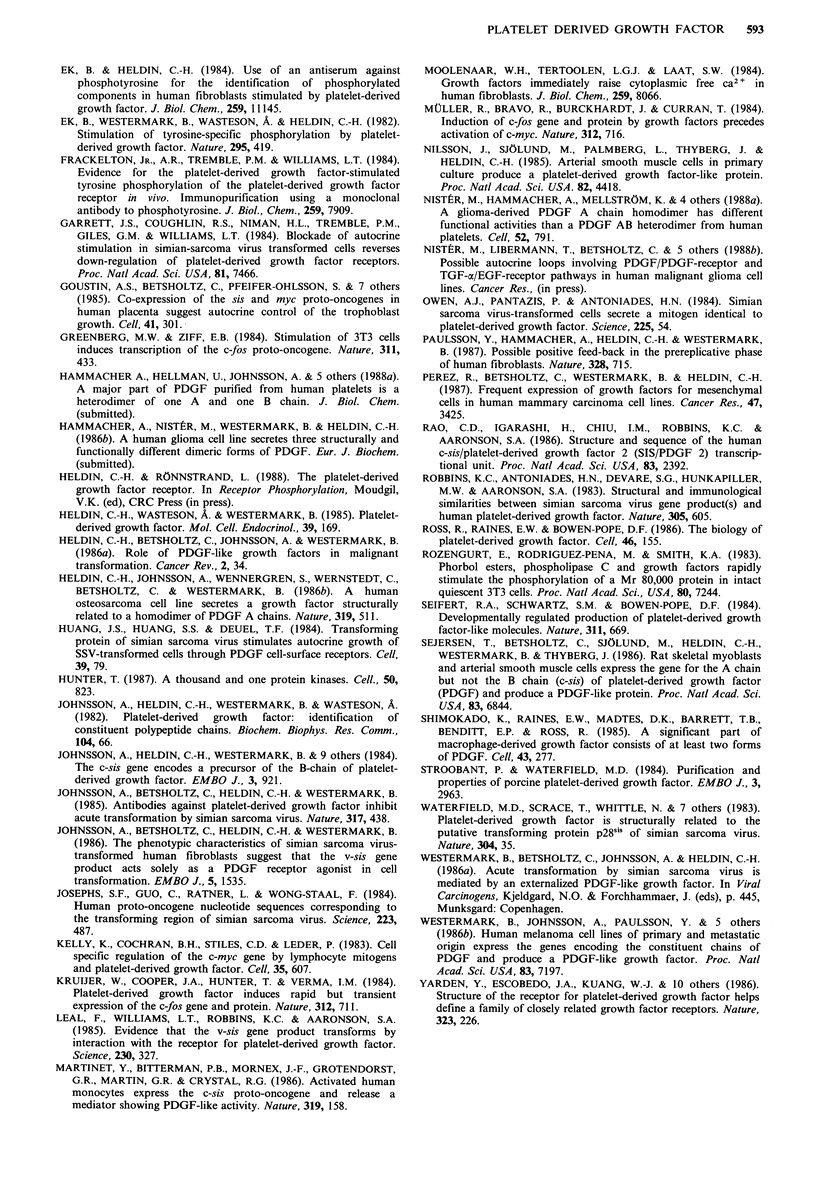# Structural and functional aspects of platelet-derived growth factor.

**DOI:** 10.1038/bjc.1988.134

**Published:** 1988-06

**Authors:** C. H. Heldin, A. Hammacher, M. NistÃ©r, B. Westermark

**Affiliations:** Ludwig Institute for Cancer Research, Uppsala, Sweden.

## Abstract

The finding that PDGF production is common in normal as well as transformed cells indicate that PDGF has a function in autocrine and paracrine stimulation of cells in several physiological and pathological conditions. The expression of mRNA for the two chains of PDGF are independently regulated. The fact that the different dimeric forms of PDGF have different functional effects, indicate that the cellular response to PDGF may be more complex than previously thought, and may involve binding to more than one receptor. The cellular effects ascribed to PDGF, growth stimulation, ruffling and chemotaxis, seems to be mainly associated with B chain containing dimers. The function of PDGF-AA remains to be elucidated.


*Br. J. Cancer* (1988), **57**, 591–593
© The Macmillan Press Ltd., 1988

### Structural and functional aspects of platelet-derived growth factor*

C.-H. Heldin[1], A. Hammacher[1], M. Nistér[2] and B. Westermark[2]

[1]*Ludwig Institute for Cancer Research, Box 595, Biomedical Center, S-751 23 Uppsala and* [2]*Department of Pathology, University Hospital, S-751 85 Uppsala, Sweden.*

**Summary** The finding that PDGF production is common in normal as well as transformed cells indicate that PDGF has a function in autocrine and paracrine stimulation of cells in several physiological and pathological conditions. The expression of mRNA for the two chains of PDGF are independently regulated. The fact that the different dimeric forms of PDGF have different functional effects, indicate that the cellular response to PDGF may be more complex than previously thought, and may involve binding to more than one receptor. The cellular effects ascribed to PDGF, growth stimulation, ruffling and chemotaxis, seems to be mainly associated with B chain containing dimers. The function of PDGF-AA remains to be elucidated.

Platelet-derived growth factor (PDGF) is a major mitogen for connective tissue cells and glial cells cultured *in vitro* (for reviews see Heldin *et al.*, 1985; Ross *et al.*, 1986). Its *in vivo* function has not been elucidated, but is localization to the blood platelets in combination with the facts that PDGF stimulates not only proliferation, but also matrix production and chemotaxis of connective tissue cells, has led to the assumption that PDGF has a role in wound healing. However, recent findings that PDGF does not only occur in platelets, but is produced by a variety of different normal and transformed cell types, indicate that PDGF may have wider functions, mediating cell proliferation in a number of normal and pathological conditions.

PDGF exerts its mitogenic effect via binding to a specific 170–185 kD cell surface receptor (reviewed in Heldin & Rönnstrand, 1988). The receptor is a transmembrane protein with an external ligand binding domain, and an internal domain with a protein tyrosine kinase activity that becomes activated after ligand binding (Ek *et al.*, 1982; Yarden *et al.*, 1986).

The mechanism whereby the mitogenic signal is transmitted from the activated receptor and further into the cell, is largely unknown. The fact that several growth factor receptors and oncogene products are protein tyrosine kinases (Hunter, 1987), indicates that tyrosine phosphorylation of specific substrates is important in stimulation of cell growth. In spite of extensive investigations (Cooper *et al.*, 1982; Ek & Heldin, 1984; Frackelton *et al.*, 1984), no substrate for the PDGF receptor kinase, with a proven role in the mitogenic pathway has yet been found. Other signals that have been discussed as part of the PDGF-stimulated mitogenic pathway, include turnover of phosphatidyl-inositol, with subsequent elevation of the cytoplasmic $Ca^{2+}$ concentration (Moolenaar *et al.*, 1984), and stimulation of protein kinase C (Rozengurt *et al.*, 1983), as well as induction of specific genes (Cochran *et al.*, 1983, Kelly *et al.*, 1983; Kruijer *et al.*, 1984; Müller *et al.*, 1984, Greenberg & Ziff, 1984).

This review will focus on structural and functional aspects of PDGF-like factors, as well as their possible involvement in autocrine or paracrine stimulation of cell growth.

### *Structures of PDGF*

PDGF is a 30 kD dimeric molecule, composed of two disulphide-bonded polypeptide chains denoted A and B (Johnsson *et al.*, 1984). The PDGF B chain is almost identical to $p^{28sis}$, the transforming protein of simian sarcoma virus (SSV) (Waterfield *et al.*, 1983; Doolittle *et al.*, 1983; see further below). Analyses of cDNAs of the A (Betsholtz *et al.*, 1986*b*) and the B (Josephs *et al.*, 1984; Collins *et al.*, 1985: Rao *et al.*, 1986) chain of PDGF revealed that both are synthesized as precursor molecules with hydrophobic signal sequences indicating that they are secretory products. The two chains, which are proteolytically processed after dimerization, are homologous to each other; within the mature parts the amino acid sequence similarity is 60% (Figure 1) (Johnsson *et al.*, 1984; Betsholtz *et al.*, 1986*b*).

**Figure 1** Schematic illustration of the different dimeric forms of PDGF. Both the A and B chains are synthesized as precursor molecules (upper part of the figure) which undergo proteolytic processing (arrows). Amino acid sequence identities are marked by black colour. The two polypeptide chains are assembled into homodimers (PDGF-AA or PDGF-BB) or heterodimers (PDGF-AB) (lower part of the figure). The dimers are held together by disulphide bonds. Note, that it is not known exactly which cysteine residues that are involved in inter-chain disulphide bonds.

Evidence was recently obtained that a major part of PDGF purified from human platelets is PDGF-AB, i.e. a heterodimer of one A chain and one B chain (Hammacher *et al.*, 1988*a*). PDGF purified from porcine platelets (Stroobant & Waterfield, 1984), as well as the transforming protein of SSV (Robbins *et al.*, 1983) have been identified as PDGF-BB. Finally, structural analyses of PDGF-like factors purified from the conditioned media of human osteosarcoma (Heldin *et al.*, 1986*b*) melanoma (Westermark *et al.*, 1986*b*) and glioma (Hammacher *et al.*, 1986*b*) cell lines, revealed the existence also of PDGF-AA. Thus, all possible dimeric combinations of PDGF chains have been found (Figure 1).

### *SSV-transformation is exerted by externalized PDGF-BB*

The structural homology between $p28^{sis}$ and PDGF (Waterfield *et al.*, 1983; Doolittle *et al.*, 1983; Robbins *et al.*, 1983), led to the hypothesis that a PDGF-like growth factor is involved in SSV-transformation. This hypothesis has received support from several subsequent observations. First, SSV-transformed cells *in vitro* produce PDGF-like growth factors which bind to and activate the PDGF receptor (Deuel *et al.*, 1983; Bowen-Pope *et al.*, 1984; Owen *et al.*, 1984; Garrett *et al.*, 1984; Huang *et al.*, 1984; Johnsson *et al.*, 1985). Second, only cell types that respond to PDGF, i.e. have PDGF receptors, can be transformed by SSV (Deinhardt, 1980; Leal *et al.*, 1985). Finally, SSV-transformation can be reverted by agents that prevent the binding

Correspondence: C.-H. Heldin.

*Presented, by invitation, at the BACR/CRC/ICRF Symposium on 'Growth factors', London, December 1987.

of PDGF to its receptor, e.g. PDGF antibodies (Johnsson *et al.*, 1985) and suramin (Betsholtz *et al.*, 1986*a*). The morphological and functional characteristics of acutely SSV-transformed human fibroblasts indicate that the infected cell receives a powerful autocrine stimulation to grow, but other features that have been associated with the malignant phenotype, e.g. immortalization, do not occur (Johnsson *et al.*, 1986). In conclusion, studies on SSV-transformed cells *in vitro* are fully compatible with a simplistic model for SSV-transformation, involving the autocrine action of a factor similar to PDGF-BB. However, to explain the malignant glioblastomas and fibrosarcomas that develop in marmoset monkeys infected by SSV (Deinhardt, 1980), one has to propose that additional genetic alterations, which are responsible for the development of the fully malignant phenotype, occur in the infected cells (Westermark *et al.*, 1986*a*).

*Production of PDGF in human tumour cells*

Expression of PDGF A or B chain mRNA, or production of PDGF receptor competing activity, have been found in a variety of human tumour cell lines (reviewed in Heldin *et al.*, 1986*a*). No correlation has been found between the expressions of the A and B chains, indicating that they are independently regulated.

Several cell lines of connective tissue cell origin or glial origin have been found to produce PDGF. Since the normal counterparts of these cells respond to PDGF, it is possible that the endogenous growth factor production has an autocrine effect and drives tumour cell growth. PDGF production is common in human glioma cell lines; 23 and 17 cell lines, out of 23 investigated, express A and B chain mRNA, respectively (Nistér *et al.*, 1988*b*).

Cell lines derived from cell types that do not respond to PDGF have also been found to produce PDGF. Since these tumour cells lack PDGF receptors it is highly unlikely that the endogenous PDGF production has any impact on tumour cell growth. PDGF production is common also in these tumour types; 8 and 9 mammary carcinoma cell lines, out of 10 investigated, were found to express the A and B chain mRNA, respectively (Perez *et al.*, 1987). If PDGF production also occurs in carcinomas *in vivo*, it is possible that this contributes to the stimulation of stroma cell proliferation, which is a common finding in many carcinomas.

*PDGF in autocrine and paracrine stimulation of normal cells*

Smooth muscle cells (Seifert *et al.*, 1984; Nilsson *et al.*, 1985) and placental cytotrophoblasts (Goustin *et al.*, 1985) have been found to produce PDGF. Since these cell types also respond to PDGF, it is possible that the PDGF production serves an autocrine function. This suggests a role of PDGF in pathophysiological reactions in the vessel wall and in placental growth. Clearly such autocrine systems in normal cells must be subjected to regulation, but little is known about the factors that exert such control functions. It was recently found that stimulation of human fibroblasts by mitogens led to expression of PDGF A chain mRNA and production of PDGF receptor competing activity (Paulsson *et al.*, 1987). Though the interpretation of this finding is not clear, it suggests the presence of a positive feed-back mechanism which may amplify the mitogenic signal.

Endothelial cells (DiCorleto & Bowen-Pope, 1983) and activated macrophages (Shimokado *et al.*, 1985; Martinet *et al.*, 1986) are examples of normal cells that produce PDGF but do not have PDGF receptors. Though it is unlikely that the PDGF production in these cases has any autocrine function, it is possible that the factor stimulates neighbouring cells in a paracrine fashion. Thus, PDGF secreted by regenerating endothelial cells in the vessel wall could stimulate the proliferation of underlying smooth muscle cells, and PDGF secreted by activated macrophages could be involved in the stimulation of connective tissue cells that is often seen in chronic inflammatory processes.

*Difference in the functional activities of different dimeric forms of PDGF*

PDGF-like growth factors were recently purified from the conditioned medium of a human glioma cell line, U–343 MGa Cl 2:6 (Hammacher *et al.*, 1986*b*), which expresses both PDGF A and B chain mRNA (Betsholtz *et al.*, 1986*b*). All three possible dimeric forms of PDGF were identified, but PDGF-AA was by far the predominant species. Analyses of PDGF-AA in several functional assays revealed differences compared to PDGF-AB; PDGF-AA had lower mitogenic activity, lower ability to stimulate actin reorganization and membrane ruffling, and no chemotactic activity (Nistér *et al.*, 1988*a*). Receptor binding experiments suggested that there exists more than one PDGF receptor class. The observed dissimilarities in functional activities of different dimeric forms of PDGF, might be explained by differences in ligand binding specificity between these receptor classes.

We thank Linda Baltell for valuable help in the preparation of this manuscript.

**References**

BETSHOLTZ, C., JOHNSSON, A., HELDIN, C.-H. & WESTERMARK, B. (1986*a*). Efficient reversion of SSV-transformation and inhibition of growth factor-induced mitogenesis by suramin. *Proc. Natl Acad. Sci. USA*, **83**, 6440.

BETSHOLTZ, C., JOHNSSON, A., HELDIN, C.-H. & 9 others (1986*b*). cDNA sequence and chromosomal localization of human platelet-derived growth factor A-chain and its expression in tumour cell lines. *Nature*, **320**, 695.

BOWEN-POPE, D.F., VOGEL, A. & ROSS, R. (1984). Production of platelet-derived growth factor-like molecules and reduced expression of platelet-derived growth factor receptors accompany transformation by a wide spectrum of agents. *Proc. Natl Acad. Sci. USA*, **81**, 2396.

COCHRAN, B.H., REFFEL, A.C. & STILES, C.D. (1983). Molecular cloning of gene sequences by regulated platelet-derived growth factor. *Cell*, **33**, 939.

COLLINS, T., GINSBURG, D., BOSS, J.M., ORKIN, S.H. & POBER, J.S. (1985). Cultured human endothelial cells express platelet-derived growth factor B chain: cDNA cloning and structural analysis. *Nature*, **316**, 748.

COOPER, J.A., BOWEN-POPE, D.F., RAINES, E., ROSS, R. & HUNTER, T. (1982). Similar effects of platelet-derived growth factor and epidermal growth factor on the phosphorylation of tyrosine in cellular proteins. *Cell*, **31**, 263.

DICORLETO, P.E. & BOWEN-POPE, D.F. (1983). Cultured endothelial cells produce a platelet-derived growth factor-like protein. *Proc. Natl Acad. Sci. USA*, **80**, 1919.

DEINHART, F. (1980). The biology of primate retrovirus. In: Klein G. (ed). *Viral Oncology*. p. 359. New York: Raven Press.

DEUEL, T.F., HUANG, J.S., HUANG, S.S. STROOBANT, P. & WATERFIELD, M.D. (1983). Expression of a platelet-derived growth factor-like protein in simian sarcoma virus transformed cells. *Science*, **221**, 1348.

DOOLITTLE, T.F., HUNKAPILLER, M.W., HOOD, L.E. & 4 others (1983), Simian sarcoma virus oncogene, v-*sis*, is derived from the gene (or genes) encoding a platelet-derived growth factor. *Science*, **221**, 275.

EK, B. & HELDIN, C.-H. (1984). Use of an antiserum against phosphotyrosine for the identification of phosphorylated components in human fibroblasts stimulated by platelet-derived growth factor. *J. Biol. Chem.*, **259**, 11145.

EK, B., WESTERMARK, B., WASTESON, Å. & HELDIN, C.-H. (1982). Stimulation of tyrosine-specific phosphorylation by platelet-derived growth factor. *Nature*, **295**, 419.

FRACKELTON, JR., A.R., TREMBLE, P.M. & WILLIAMS, L.T. (1984). Evidence for the platelet-derived growth factor-stimulated tyrosine phosphorylation of the platelet-derived growth factor receptor *in vivo*. Immunopurification using a monoclonal antibody to phosphotyrosine. *J. Biol., Chem.*, **259**, 7909.

GARRETT, J.S., COUGHLIN, R.S., NIMAN, H.L., TREMBLE, P.M., GILES, G.M. & WILLIAMS, L.T. (1984). Blockade of autocrine stimulation in simian-sarcoma virus transformed cells reverses down-regulation of platelet-derived growth factor receptors. *Proc. Natl Acad. Sci. USA*, **81**, 7466.

GOUSTIN, A.S., BETSHOLTZ, C., PFEIFER-OHLSSON, S. & 7 others (1985). Co-expression of the *sis* and *myc* proto-oncogenes in human placenta suggest autocrine control of the trophoblast growth. *Cell*, **41**, 301.

GREENBERG, M.W. & ZIFF, E.B. (1984). Stimulation of 3T3 cells induces transcription of the c-*fos* proto-oncogene. *Nature*, **311**, 433.

HAMMACHER A., HELLMAN, U., JOHNSSON, A. & 5 others (1988*a*). A major part of PDGF purified from human platelets is a heterodimer of one A and one B chain. *J. Biol. Chem.* (submitted).

HAMMACHER, A., NISTÉR, M., WESTERMARK, B. & HELDIN, C.-H. (1986*b*). A human glioma cell line secretes three structurally and functionally different dimeric forms of PDGF. *Eur. J. Biochem.* (submitted).

HELDIN, C.-H. & RÖNNSTRAND, L. (1988). The platelet-derived growth factor receptor. In *Receptor Phosphorylation*, Moudgil, V.K. (ed), CRC Press (in press).

HELDIN, C.-H., WASTESON, Å. & WESTERMARK, B. (1985). Platelet-derived growth factor. *Mol. Cell. Endocrinol.*, **39**, 169.

HELDIN, C.-H., BETSHOLTZ, C., JOHNSSON, A. & WESTERMARK, B. (1986*a*). Role of PDGF-like growth factors in malignant transformation. *Cancer Rev.*, **2**, 34.

HELDIN, C.-H., JOHNSSON, A., WENNERGREN, S., WERNSTEDT, C., BETSHOLTZ, C. & WESTERMARK, B. (1986*b*). A human osteosarcoma cell line secretes a growth factor structurally related to a homodimer of PDGF A chains. *Nature*, **319**, 511.

HUANG, J.S., HUANG, S.S. & DEUEL, T.F. (1984). Transforming protein of simian sarcoma virus stimulates autocrine growth of SSV-transformed cells through PDGF cell-surface receptors. *Cell*, **39**, 79.

HUNTER, T. (1987). A thousand and one protein kinases. *Cell.*, **50**, 823.

JOHNSSON, A., HELDIN, C.-H., WESTERMARK, B. & WASTESON, Å. (1982). Platelet-derived growth factor: identification of constituent polypeptide chains. *Biochem. Biophys. Res. Comm.*, **104**, 66.

JOHNSSON, A., HELDIN, C.-H., WESTERMARK, B. & 9 others (1984). The c-*sis* gene encodes a precursor of the B-chain of platelet-derived growth factor. *EMBO J.*, **3**, 921.

JOHNSSON, A., BETSHOLTZ, C., HELDIN, C.-H. & WESTERMARK, B. (1985). Antibodies against platelet-derived growth factor inhibit acute transformation by simian sarcoma virus. *Nature*, **317**, 438.

JOHNSSON, A., BETSHOLTZ, C., HELDIN, C.-H. & WESTERMARK, B. (1986). The phenotypic characteristics of simian sarcoma virus-transformed human fibroblasts suggest that the v-*sis* gene product acts solely as a PDGF receptor agonist in cell transformation. *EMBO J.*, **5**, 1535.

JOSEPHS, S.F., GUO, C., RATNER, L. & WONG-STAAL, F. (1984). Human proto-oncogene nucleotide sequences corresponding to the transforming region of simian sarcoma virus. *Science*, **223**, 487.

KELLY, K., COCHRAN, B.H., STILES, C.D. & LEDER, P. (1983). Cell specific regulation of the c-*myc* gene by lymphocyte mitogens and platelet-derived growth factor. *Cell*, **35**, 607.

KRUIJER, W., COOPER, J.A., HUNTER, T. & VERMA, I.M. (1984). Platelet-derived growth factor induces rapid but transient expression of the c-*fos* gene and protein. *Nature*, **312**, 711.

LEAL, F., WILLIAMS, L.T., ROBBINS, K.C. & AARONSON, S.A. (1985). Evidence that the v-*sis* gene product transforms by interaction with the receptor for platelet-derived growth factor. *Science*, **230**, 327.

MARTINET, Y., BITTERMAN, P.B., MORNEX, J.-F., GROTENDORST, G.R., MARTIN, G.R. & CRYSTAL, R.G. (1986). Activated human monocytes express the c-*sis* proto-oncogene and release a mediator showing PDGF-like activity. *Nature*, **319**, 158.

MOOLENAAR, W.H., TERTOOLEN, L.G.J. & LAAT, S.W. (1984). Growth factors immediately raise cytoplasmic free $ca^{2+}$ in human fibroblasts. *J. Biol. Chem.*, **259**, 8066.

MÜLLER, R., BRAVO, R., BURCKHARDT, J. & CURRAN, T. (1984). Induction of c-*fos* gene and protein by growth factors precedes activation of c-*myc*. *Nature*, **312**, 716.

NILSSON, J., SJÖLUND, M., PALMBERG, L., THYBERG, J. & HELDIN, C.-H. (1985). Arterial smooth muscle cells in primary culture produce a platelet-derived growth factor-like protein. *Proc. Natl Acad. Sci. USA*. **82**, 4418.

NISTÉR, M., HAMMACHER, A., MELLSTRÖM, K. & 4 others (1988*a*). A glioma-derived PDGF A chain homodimer has different functional activities than a PDGF AB heterodimer from human platelets. *Cell*, **52**, 791.

NISTÉR, M., LIBERMANN, T., BETSHOLTZ, C. & 5 others (1988*b*). Possible autocrine loops involving PDGF/PDGF-receptor and TGF-α/EGF-receptor pathways in human malignant glioma cell lines. *Cancer Res.*, (in press).

OWEN, A.J., PANTAZIS, P. & ANTONIADES, H.N. (1984). Simian sarcoma virus-transformed cells secrete a mitogen identical to platelet-derived growth factor. *Science*, **225**, 54.

PAULSSON, Y., HAMMACHER, A., HELDIN, C.-H. & WESTERMARK, B. (1987). Possible positive feed-back in the prereplicative phase of human fibroblasts. *Nature*, **328**, 715.

PEREZ, R., BETSHOLTZ, C., WESTERMARK, B. & HELDIN, C.-H. (1987). Frequent expression of growth factors for mesenchymal cells in human mammary carcinoma cell lines. *Cancer Res.*, **47**, 3425.

RAO, C.D., IGARASHI, H., CHIU, I.M., ROBBINS, K.C. & AARONSON, S.A. (1986). Structure and sequence of the human c-*sis*/platelet-derived growth factor 2 (SIS/PDGF 2) transcriptional unit. *Proc. Natl Acad. Sci. USA*, **83**, 2392.

ROBBINS, K.C., ANTONIADES, H.N., DEVARE, S.G., HUNKAPILLER, M.W. & AARONSON, S.A. (1983). Structural and immunological similarities between simian sarcoma virus gene product(s) and human platelet-derived growth factor. *Nature*, **305**, 605.

ROSS, R., RAINES, E.W. & BOWEN-POPE, D.F. (1986). The biology of platelet-derived growth factor. *Cell*, **46**, 155.

ROZENGURT, E., RODRIGUEZ-PENA, M. & SMITH, K.A. (1983). Phorbol esters, phospholipase C and growth factors rapidly stimulate the phosphorylation of a Mr 80,000 protein in intact quiescent 3T3 cells. *Proc. Natl Acad. Sci., USA*, **80**, 7244.

SEIFERT, R.A., SCHWARTZ, S.M. & BOWEN-POPE, D.F. (1984). Developmentally regulated production of platelet-derived growth factor-like molecules. *Nature*, **311**, 669.

SEJERSEN, T., BETSHOLTZ, C., SJÖLUND, M., HELDIN, C.-H., WESTERMARK, B. & THYBERG, J. (1986). Rat skeletal myoblasts and arterial smooth muscle cells express the gene for the A chain but not the B chain (c-*sis*) of platelet-derived growth factor (PDGF) and produce a PDGF-like protein. *Proc. Natl Acad. Sci. USA*, **83**, 6844.

SHIMOKADO, K., RAINES, E.W., MADTES, D.K., BARRETT, T.B., BENDITT, E.P. & ROSS, R. (1985). A significant part of macrophage-derived growth factor consists of at least two forms of PDGF. *Cell*, **43**, 277.

STROOBANT, P. & WATERFIELD, M.D. (1984). Purification and properties of porcine platelet-derived growth factor. *EMBO J.*, **3**, 2963.

WATERFIELD, M.D., SCRACE, T., WHITTLE, N. & 7 others (1983). Platelet-derived growth factor is structurally related to the putative transforming protein $p28^{sis}$ of simian sarcoma virus. *Nature*, **304**, 35.

WESTERMARK, B., BETSHOLTZ, C., JOHNSSON, A. & HELDIN, C.-H. (1986*a*). Acute transformation by simian sarcoma virus is mediated by an externalized PDGF-like growth factor. In *Viral Carcinogens*, Kjeldgard, N.O. & Forchhammaer, J. (eds), p. 445, Munksgard: Copenhagen.

WESTERMARK, B., JOHNSSON, A., PAULSSON, Y. & 5 others (1986*b*). Human melanoma cell lines of primary and metastatic origin express the genes encoding the constituent chains of PDGF and produce a PDGF-like growth factor. *Proc. Natl Acad. Sci. USA*, **83**, 7197.

YARDEN, Y., ESCOBEDO, J.A., KUANG, W.-J. & 10 others (1986). Structure of the receptor for platelet-derived growth factor helps define a family of closely related growth factor receptors. *Nature*, **323**, 226.